# Supplementary material for: Long-range inhibition from prelimbic to cingulate areas of the medial prefrontal cortex enhances network activity and response execution
Source: Nat Commun. 2024 Jul 10;15:5772. doi: 10.1038/s41467-024-50055-z (PMC11233578; doi:10.1038/s41467-024-50055-z)
Supplement: Supplementary file 1 — Supplementary Information [file 41467_2024_50055_MOESM1_ESM.pdf]

Supplementary information for

Long-range inhibition from prelimbic to cingulate areas of the medial prefrontal cortex enhances network activity and response execution

Nao Utashiro[1,4], Duncan Archibald Allan MacLaren[1,4], Yu-Chao Liu[1,4], Kaneschka Yaqubi[1,2], Birgit Wojak[1,3], Hannah Monyer[1,5,*]

[*] Correspondence: h.monyer@dkfz-heidelberg.de

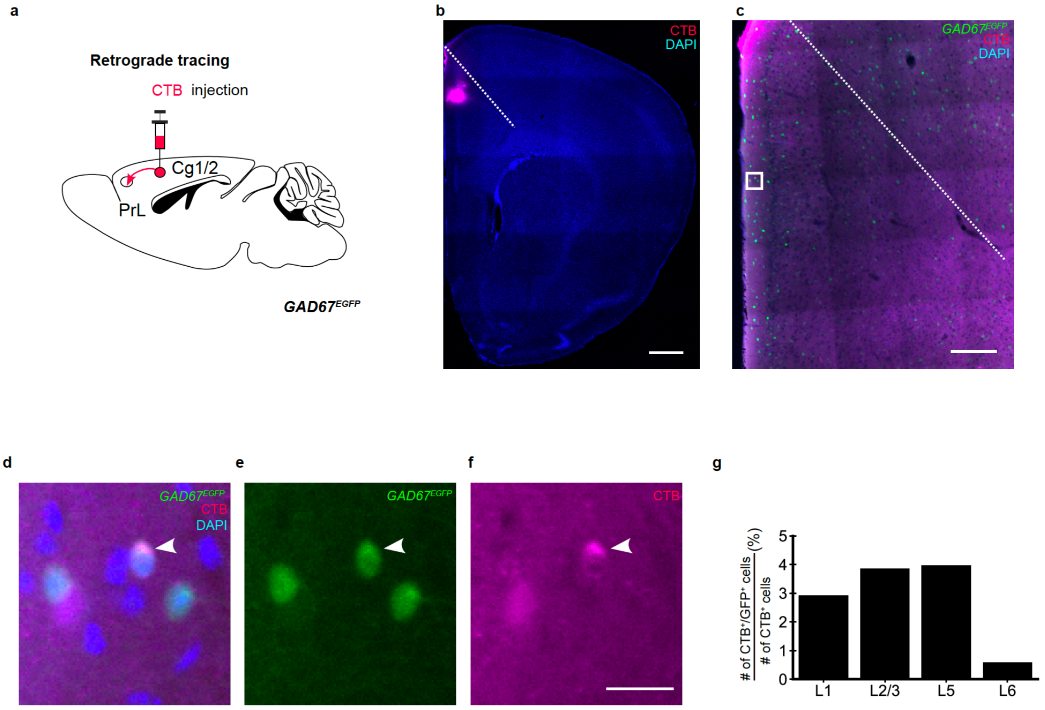

Supplementary Figure 1 | PrL GABAergic projection neurons reside in all cortical layers. **a**, Schematic of the injection for retrograde tracing experim ents. Cholera toxin subunit B conjugated Alexa Fluor 555 (CTB-Alexa 555) was injected into the Cg1/2 of *GAD67*^{EGFP} mice. **b**, Representative confocal image of a DAPI stained coronal section showing the CTB-Alexa 555 signal (magenta) at the injection site in the Cg1/2. **c**, Representative confocal image of a DAPI stained section showing retrogradely labelled CTB^{+} neurons (magenta) and GABAergic GFP^{+} neurons (green) in the PrL. **d-f**, Magnified view of the boxed area in panel (**c**) showing a CTB^{+}/GFP^{+} neuron (white arrowhead). **g**, Proportion of CTB^{+}/GFP^{+} double positive neurons in each layer of the PrL (9 slices from N = 3 mice). Scale bar in **b**, 500 µm, in **c**, 200 µm, in **d-f**, 20 µm. Abbreviation, PrL, prelimbic cortex, Cg1/2, cingulate area 1 and 2.

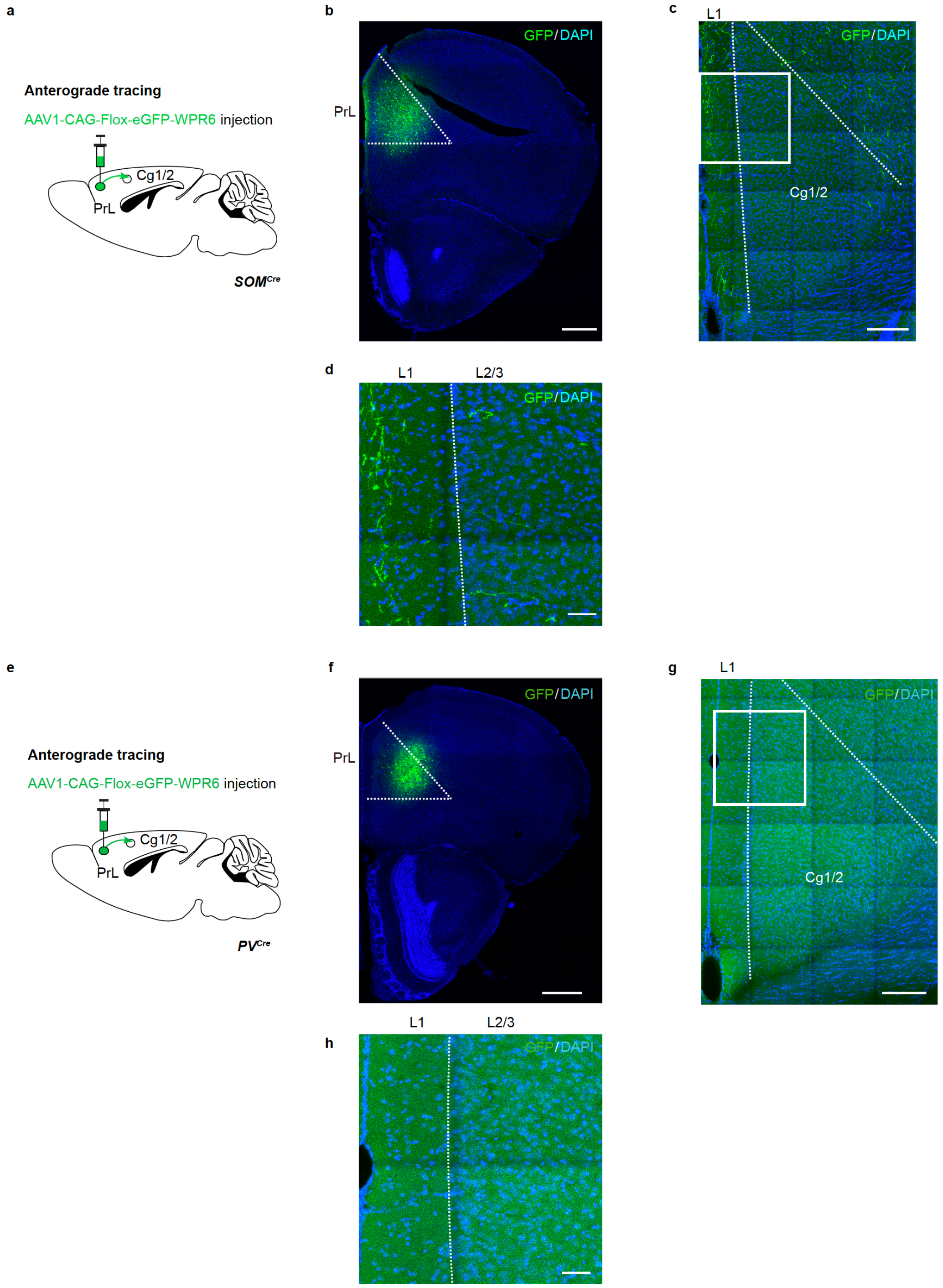

Supplementary Figure 2 | PrL GABAergic projection neurons comprise SOM^{+} neurons.

a, Schematic of the injection for anterograde tracing. AAV1-CAG-Flox-eGFP-WPR6 was injected into the PrL of *SOM*^{Cre} mice. **b**, Representative confocal image of Cre-dependent eGFP

(green) expression in GABAergic neurons at the injection site in the PrL in a DAPI stained (blue) coronal section. **c**, Representative confocal image of a DAPI stained coronal section showing PrL SOM$^+$ neuron-derived GFP$^+$ axons in the Cg1/2. **d,** Magnified view of the boxed area in panel (**c**). **e**, Schematic of the injection for anterograde tracing. AAV1-CAG-Flox-eGFP-WPR6 was injected into the PrL of PV^{Cre} mice. **f**, Representative confocal image of Cre-dependent eGFP (green) expression in GABAergic neurons at the injection site in the PrL in a DAPI stained (blue) coronal section. **g**, Representative confocal image of a DAPI stained coronal section in the Cg1/2. **h**, Magnified view of the boxed area in panel (**g**). **b-d**, Images were obtained from 3 mice and **f-h**, from 2 mice. Scale bar in **b**, **f**, 500 μm, in **c**, **g**, 200 μm, in **d**, **h**, 50 μm. Abbreviation, PrL, prelimbic cortex, Cg1/2, cingulate area 1 and 2

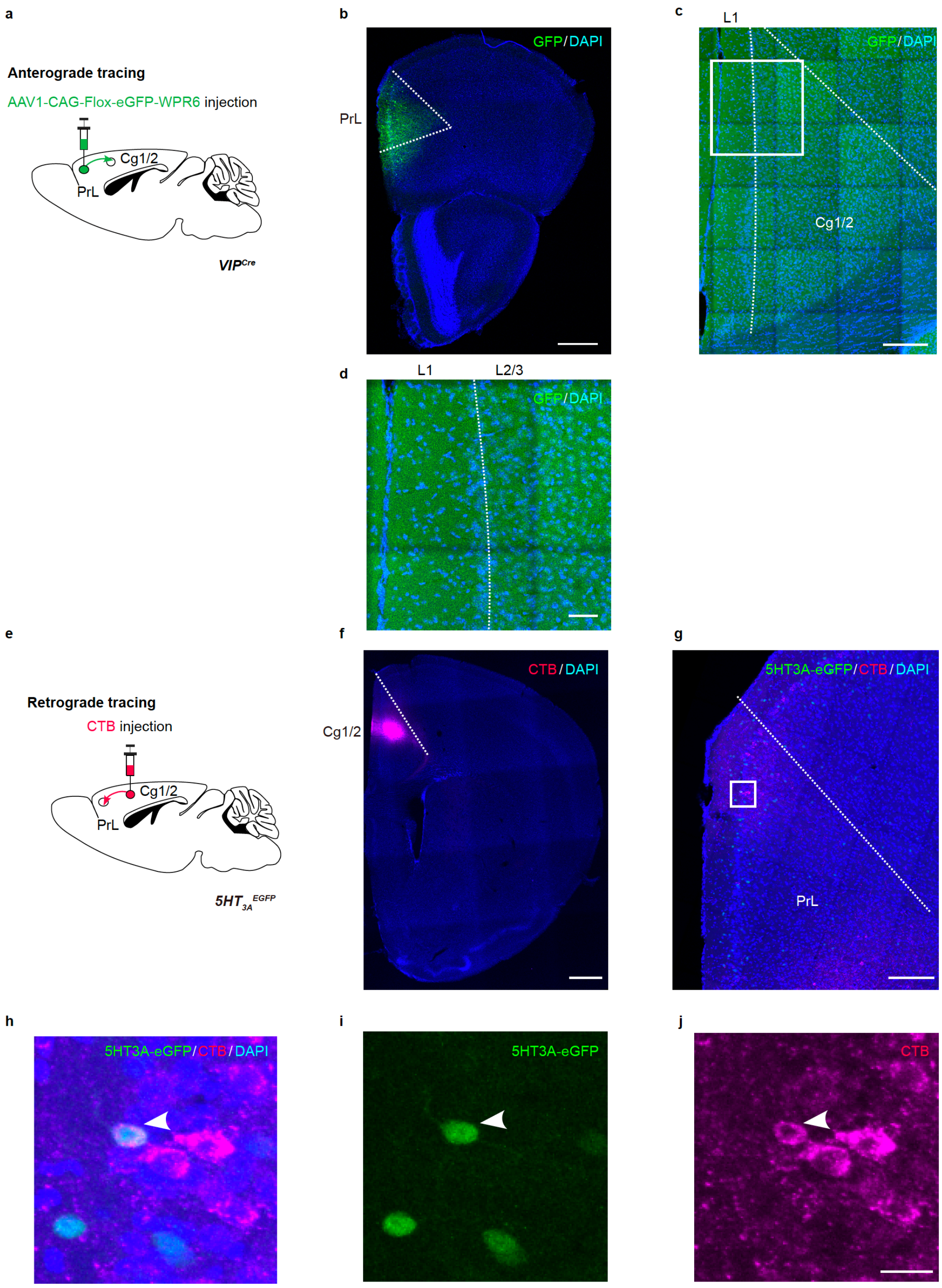

Supplementary Figure 3 | PrL GABAergic projection neurons comprise 5HT3A^{+} /VIP^{-} neurons. **a**, Schematic of the injection for anterograde tracing. AAV1-CAG-Flox-eGFP-WPR6 was injected into the PrL of *VIP*^{Cre} mice. **b**, Representative confocal image of Cre-dependent eGFP

(green) expression in GABAergic neurons at the injection site in the PrL in a DAPI stained (blue) coronal section. **c**, Representative confocal image of a DAPI stained coronal section in the Cg1/2. **d**, Magnified view of the boxed area in panel (**c**). **e**, Schematic of the injection for retrograde tracing. Cholera toxin subunit B conjugated Alexa Fluor 555 (CTB-Alexa 555) was injected into the Cg1/2 of $5HT3A^{EGFP}$ mice. **f**, Representative confocal image of a DAPI stained coronal section showing the CTB-Alexa 555 signal (magenta) at the injection site in the Cg1/2. **g**, Representative confocal image of a DAPI stained section showing retrogradely labelled CTB^{+} neurons (magenta) and GABAergic GFP^{+} neurons (green) in the PrL. **h-j**, Magnified view of the boxed area in panel (**g**) showing a CTB^{+}/GFP^{+} neuron (white arrowhead). **b-d**, Images were obtained from 5 mice. **f-j**, Images were obtained from 3 mice. Scale bar in **b**, **f**, 500 μm, in **c**, **g**, 200 μm, in **d**, 50 μm and in **h-j**, 20 μm. Abbreviation, PrL, prelimbic cortex, Cg1/2, cingulate area 1 and 2

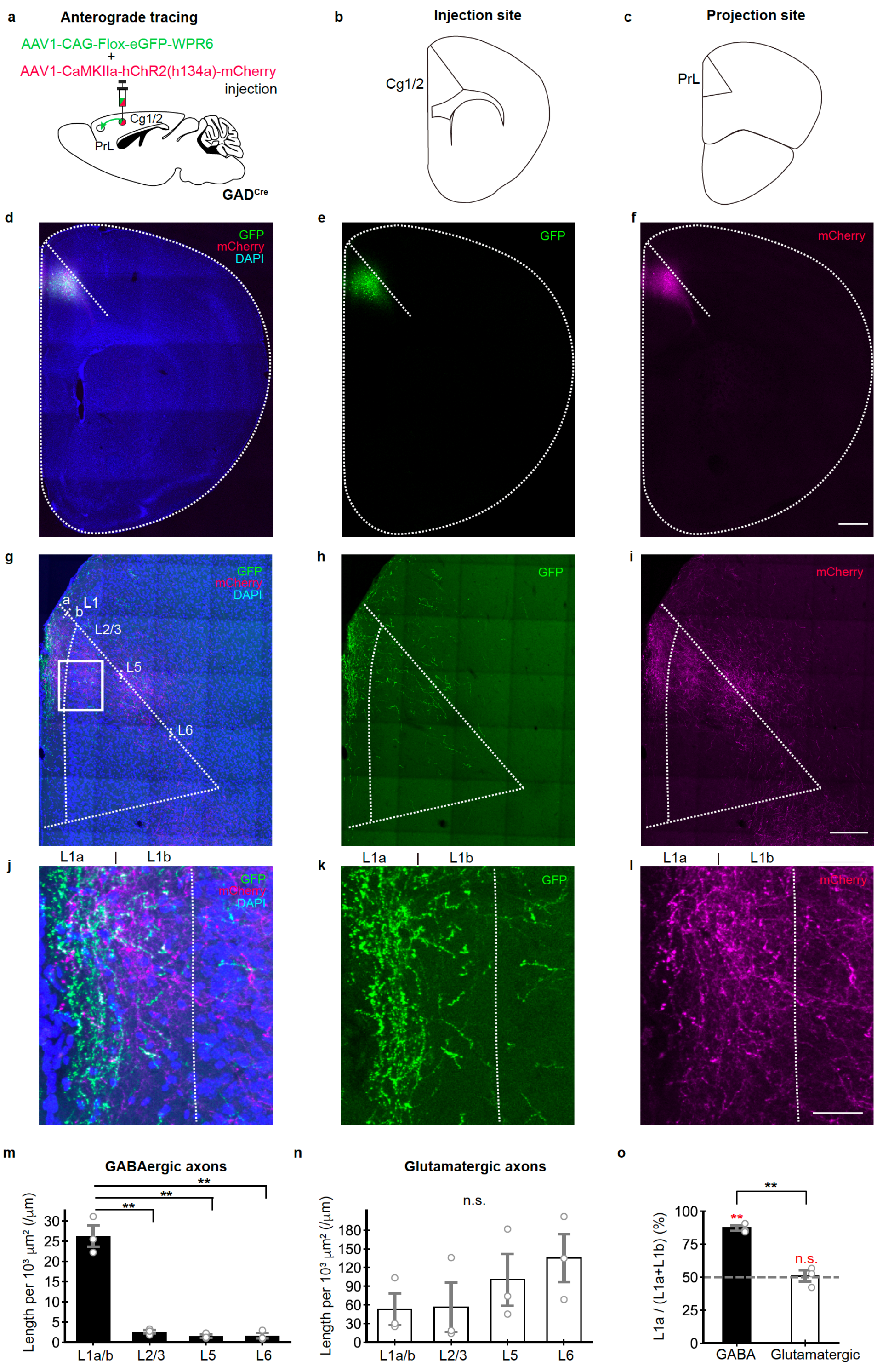

Supplementary Figure 4 | Cg1/2 GABAergic projection neurons target mainly L1 in the PrL.

a, Schematic of the injection for anterograde tracing. AAV1-CAG-Flox-eGFP-WPR6 and AAV1-CAMKIIa-hChR2(h134a)-mCherry were injected into the Cg1/2 of GAD^{Cre} mice. **b**, Schematic

of the injection site in the Cg1/2 and **c**, the projection site in the PrL in a coronal section. **d**, Representative confocal image of Cre-dependent eGFP (green) and CAMKII-promoter dependent mCherry (magenta) expression at the injection site in the Cg1/2 in a DAPI stained (blue) coronal section. **e,** Cre-dependent eGFP expression in GABAergic neurons. **f,** mCherry expression in excitatory neurons. **g**, Representative confocal image of a DAPI stained coronal section showing eGFP and mCherry at the projection site, i.e. the PrL. **h,** Cg1/2 GABAergic neuron-derived GFP expressing axons in the PrL. **i**, Cg1/2 excitatory neuron-derived mCherry expressing axons in the PrL. **j-l**, Magnified view of the boxed area in panel (**g**). **m**, Density of GABAergic axons (L1 vs. L2/3, $P = 4.02e\text{-}05$, L1 vs. L5, $P = 1.88e\text{-}07$, L1 vs. L6, $P = 1.57e\text{-}09$, DF = 8, two-sided Dunnett's test) and **n**, excitatory axons in the indicated layers of the PrL (L1 vs. L2/3, $P = 0.999$, L1 vs. L5, $P = 0.702$, L1 vs. L6, $P = 0.328$, DF = 8, two-sided Dunnett's test). Open circles represent mean values from individual mice. **o**, GABAergic and excitatory axons calculated as percentage of total axons in L1a ($P = 0.00151$, DF = 4, two-sided t-test). red asterisks and n.s. above bars refer to a one-sample t-test of the percentage of GABAergic or excitatory neuron axons in L1a versus 50% (GABAergic, $P = 0.00269$, DF = 2, glutamatergic, $P = 0.866$, DF = 2). **d-o**, Images and data were obtained from 9 slices from 3 mice. **m-o**, Data were presented as mean ± S.E.M.. Scale bar in **d-f**, 500 μm, in **g-i**, 200 μm, in **j-l**, 50 μm. Abbreviation, PrL, prelimbic cortex, Cg1/2, cingulate area 1 and 2, eGFP, enhanced green fluorescent protein, DAPI, 4',6-diamidino-2-phenylindole. n.s., not significant, $^{**}P < 0.01$, DF, degree of freedom

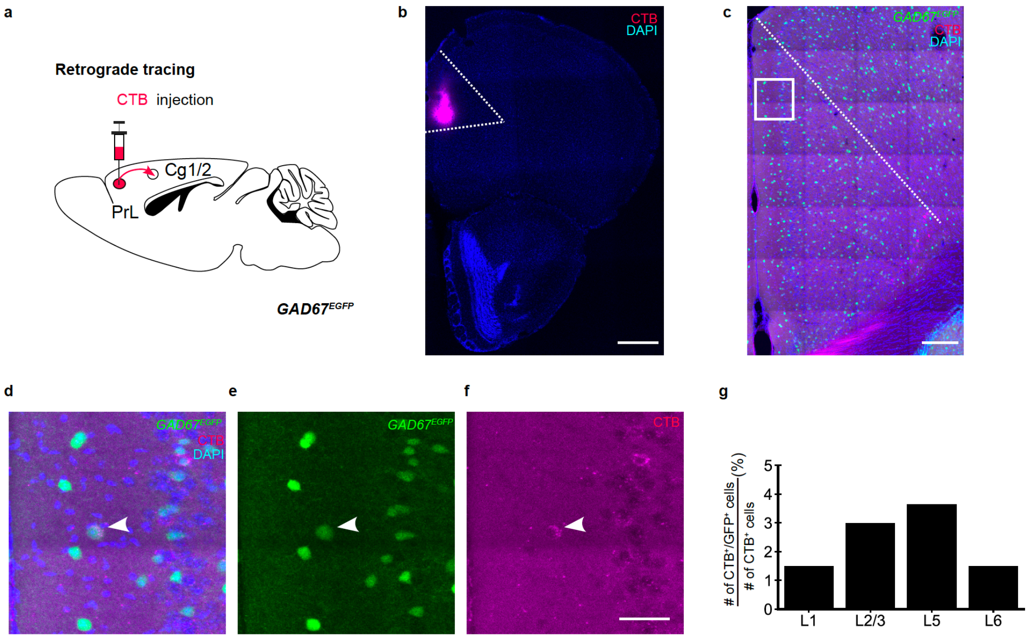

Supplementary Figure 5 | Cg1/2 GABAergic projection neurons reside in all cortical layers. **a**, Schematic of the injection for retrograde tracing. Cholera toxin subunit B conjugated Alexa Fluor 555 (CTB-Alexa 555) was injected into the PrL of *GAD67*^{EGFP} mice. **b**, Representative confocal image of a DAPI stained coronal section showing the CTB-Alexa 555 signal (magenta) at the injection site in the PrL. **c**, Representative confocal image of a DAPI stained section showing retrogradely labelled CTB^{+} neurons (magenta) and GABAergic GFP^{+} neurons (green) in the Cg1/2. **d-f**, Magnified view of the boxed area in panel (**c**) showing a CTB^{+}/GFP^{+} neuron (white arrowhead). **g**, Proportion of CTB^{+}/GFP^{+} double positive neurons in each layer of the Cg1/2 (9 slices from N = 3 mice). Scale bar in **b**, 500 μm, in **c**, 200 μm, in **d-f**, 50 μm. Abbreviation, PrL, prelimbic cortex, Cg1/2, cingulate area 1 and 2

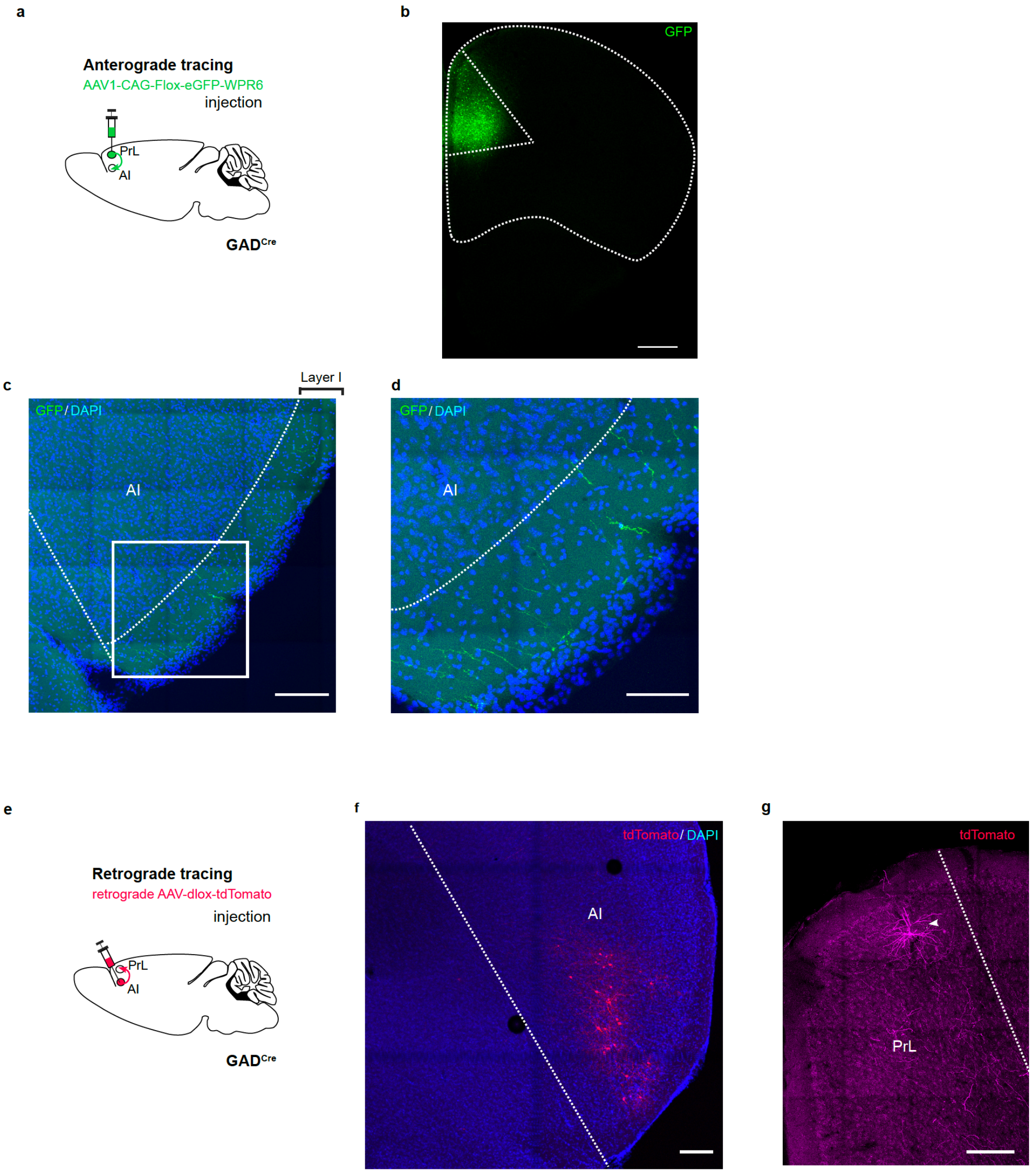

Supplementary Figure 6 | PrL GABAergic projection neurons target mainly L1 in the agranular insula (AI).

a, Schematic of the injection for anterograde tracing. AAV1-CAG-Flox-eGFP-WPR6 was injected into the PrL of GAD^{Cre} mice. **b**, Representative confocal image of Cre-dependent eGFP (green) at the injection site in the PrL in a coronal section. The image is also shown in panel (**e**) of Fig. 1. **c**, Representative confocal image of a coronal section showing PrL GABAergic neuron-derived GFP^{+} axons in the AI. **d**, Magnified view of the boxed area in panel (**c**). **e**, Schematic of

retrograde tracing. Retrograde AAV-dlox-tdTomato was injected into the AI. **f**, Representative confocal image of the injection site in the AI. **g**, Representative confocal image of a coronal section showing $tdTomato^+$ expression (magenta) in GABAergic neurons (white arrowheads) in the PrL of a mouse that had been injected with a retrograde AAV-dlox-tdTomato in the AI. **b**-**d**, **f**, **g**, Images were obtained from 3 mice. Scale bar in **b**, 500 μm, **c**, **f**, **g**, 200 μm, in **d**, 100 μm. Abbreviation, PrL, prelimbic cortex, AI, agranular insular cortex, eGFP, enhanced green fluorescent protein, DAPI, 4',6-diamidino-2-phenylindole.

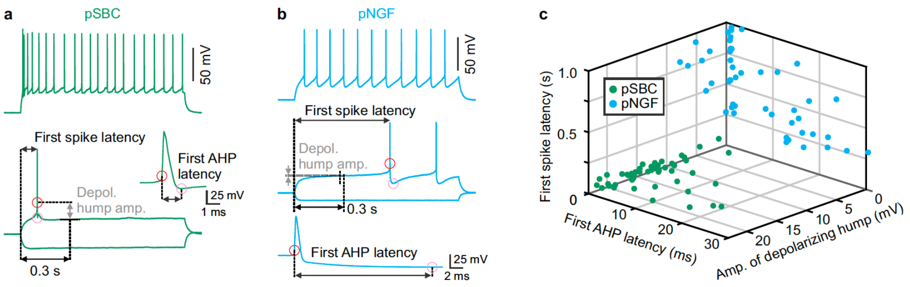

Supplementary Figure 7 | L1 INs in Cg1/2 exhibit distinct firing patterns.

a, **b**, Representative voltage responses evoked by current pulses (-50, near threshold, 100 pA) in a pSBC (**a**) and a pNGF (**b**). Note that the pSBC has a short first spike latency, a depolarizing hump and short first afterhyperpolarization (AHP) latency, whereas the pNGF has a long first spike latency and a long first AHP latency. Red and pink circles indicate action potential threshold and AHP minimum, respectively. **c**, 3D plot of first AHP latency, initial depolarizing hump amplitude (depol. hump amp.) and first spike latency for 97 L1 neurons in Cg1/2. Putative SBCs (n = 48 cells, N = 16 mice) and pNGFs (n = 49 cells, N = 15 mice) are indicated in green and blue, respectively.

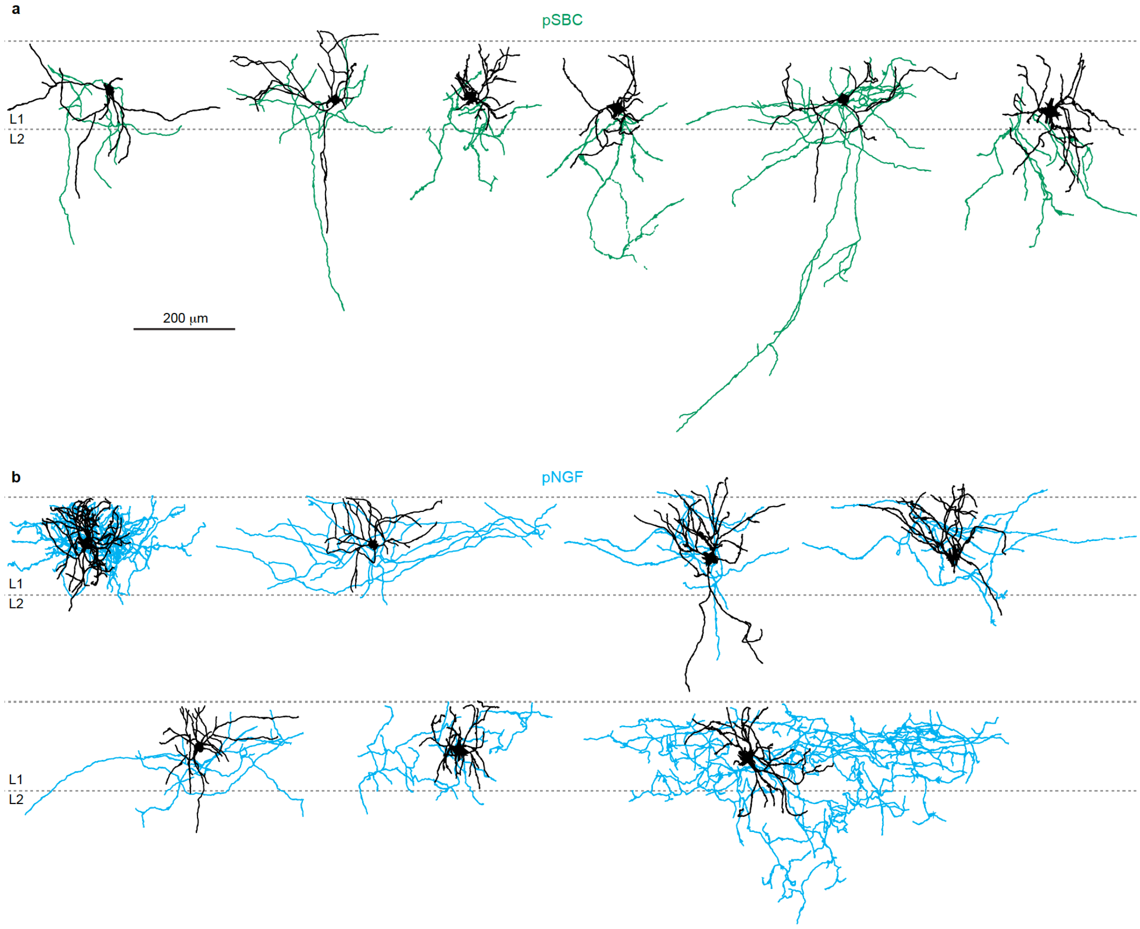

Supplementary Figure 8 | Morphological reconstruction of 13 L1 INs in the Cg1/2

a, Reconstruct of 6 pSBCs. Somata and dendrites are indicated in black. Axonal arborization is indicated in green and blue. The borders of cortical layers are indicated as dotted lines. **b**, Reconstruction of 7 pNGFs.

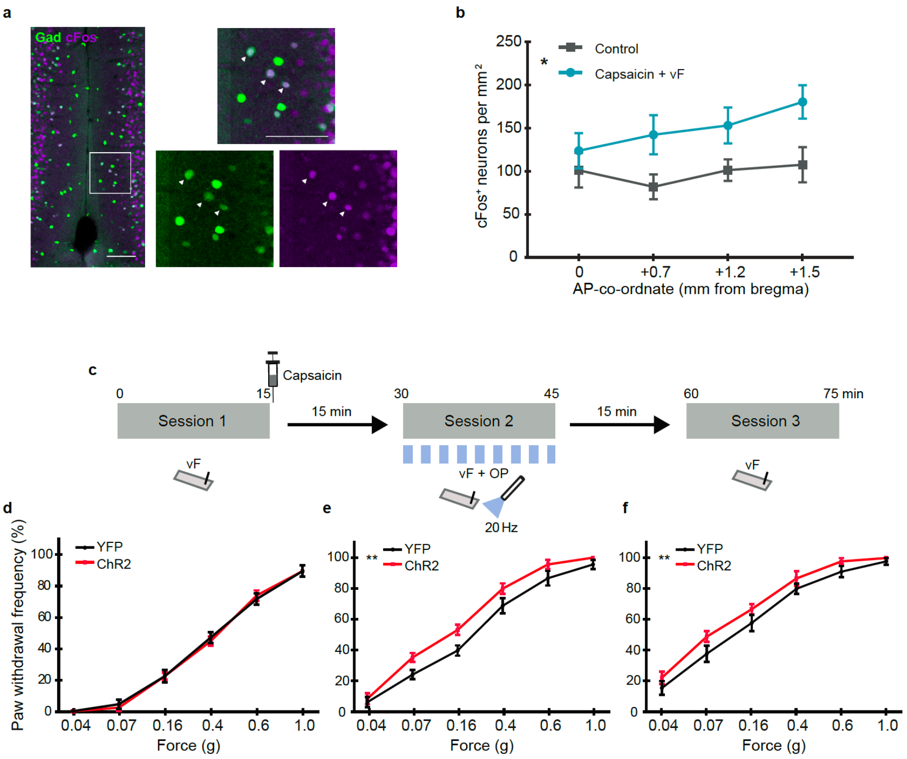

Supplementary Figure 9 | Optogenetic activation of PrL GABAergic projections in the Cg1/2 increases capsaicin-evoked mechanical hypersensitivity.

a, Left: Representative confocal image showing cFos labeling (purple) and GABAergic neurons (green) in the Cg1/2, Right: magnified views of the boxed area in the left image and split by channel, with L1 cFos$^+$ neurons indicated by white arrows. **b,** Quantification of cFos$^+$ cells in L1 of the Cg1/2 following capsaicin injection into the lower hindleg and plantar applications of von Frey filaments. There is a significant increase in the number of cFos$^+$ neurons in the capsaicin + von Frey group compared to the control group which received exposure to the testing apparatus but no capsaicin or von Frey filament applications (control N = 6, capsaicin + vF N = 6. Main effect of *group* $F(1,10) = 5.96$, $P = 0.0348$, but no *group* x *AP-co-ordinate* interaction, $P > 0.05$.). **c**, schematic of the optogenetic experimental protocol. GAD^{cre} mice were injected with virus (AAV-DIO-ChR2-mCherry, N = 9 mice, AAV-DIO-YFP, N = 9 mice) into the PrL and an optic fiber was implanted into the Cg1/2. They were subsequently tested in a mechanical

hypersensitivity assay using von Frey filaments after an injection of capsaicin into the lower hind leg (session 1, pre-optical stimulation and pre-capsaicin; session 2, post-capsaicin and peri-optical stimulation; session 3, post-capsaicin and post-optical stimulation) each with 6 different vF filament forces. **d**, there is no difference in basal mechanical sensitivity between ChR2 and YFP groups prior to optical stimulation and capsaicin treatment (all $P > 0.05$). **e**, following capsaicin injection into lower hind leg, optical stimulation of PrL GABAergic terminals increases the paw withdrawal frequency in the ChR2 group (Main effect of *group* $F(1,16) = 9.62$, $P = 0.0069$, but no gr*oup* x *force* interaction, $P > 0.05$). **f**, the enhanced responsiveness to the vF stimulation in the ChR2 group persists post-optical stimulation and capsaicin treatment (Main effect of *group* $F(1,16) = 11.11$, $P = 0.0042$, but no gr*oup* x *force* interaction $P > 0.05$). All data were analyzed by RM ANOVA followed by two-sided Bonferroni corrected multiple comparisons. Scale bars indicate 100 μm. AP, anterior-posterior. * $p < 0.05$. Lines on graphs show group means ± S.E.M..

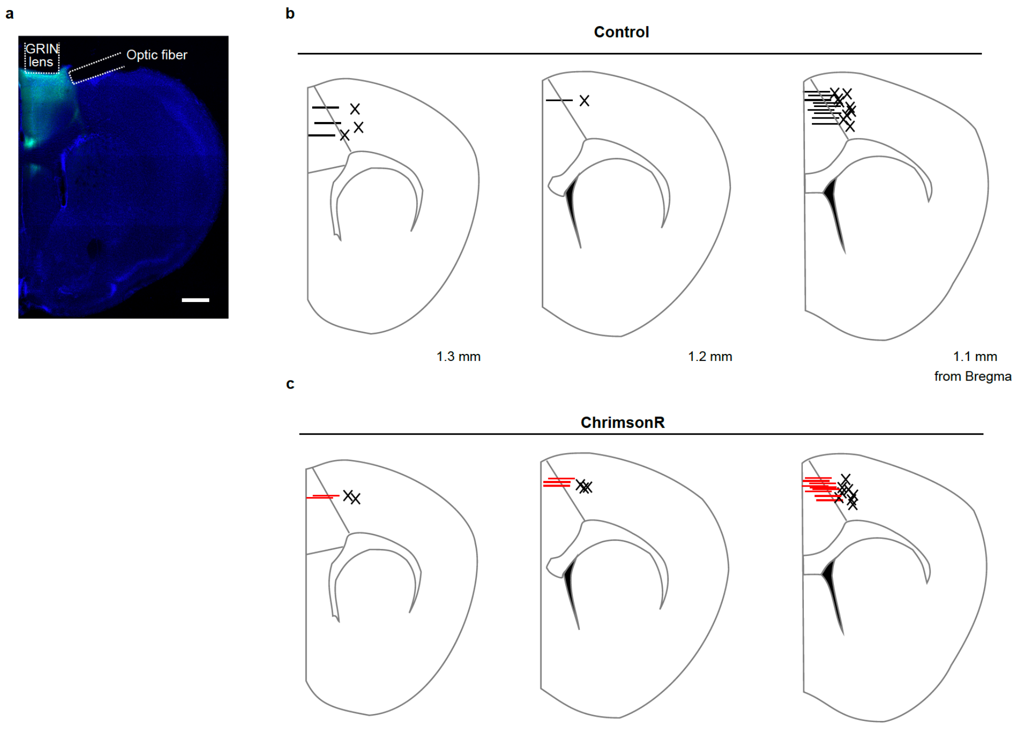

Supplementary Figure 10 | Implantation sites of GRIN lens and optic fiber in the Cg1/2.

a, Representative image showing CamKII promoter-dependent GCaMP6s expression (green) in Cg1/2 neurons in a DAPI stained (blue) coronal section with indicated GRIN lens and optic fiber placement (dashed lines). Scale bar, 500 µm. **b**, Implantation sites of GRIN lens (lines) and optic fiber tips (crosses) in all control (N = 13 mice) and, **c**, ChrimsonR (N = 15 mice) mice.

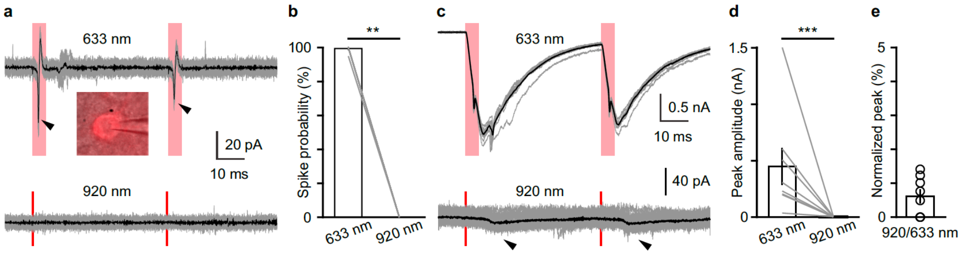

Supplementary Figure 11 | ChrimsonR-expressing neurons in PrL are effectively activated by 633 nm but not 920 nm photo illumination.

a, Top, overlay of IR-DGC and epifluorescence images of a ChrimsonR-expressing GABAergic cell in PrL. Representative traces of action currents (arrowheads) evoked by red light stimulation (633 nm, 5-ms pulses) under cell-attached configuration in the same cell. Bottom, no spiking activity detected in the same cell when scanning with two-photon laser (920 nm, same scanning settings and power used for calcium imaging). Individual traces are superimposed and shown in gray. Averaged traces are shown in black. Colored columns indicate photo illumination. **b**, Summary of spike probability (633-nm: 99.44 ± 0.56%, 920-nm: 0.00 ± 0.00%, n = 9 cells, N = 2 mice, $P = 0.0048$, two-sided Wilcoxon signed rank test). **c**, Representative traces of ChrimsonR-mediated inward currents recorded under whole-cell configuration (holding potential: -80 mV) in the same cell as in (**a**). Note that red light stimulation (633 nm) evoked prominent inward currents whereas two-photon scanning (920 nm) elicited negligible inward currents (arrowheads). **d**, Summary of ChrimsonR-mediated currents (633-nm: 448.9 ± 163.6 pA, 920-nm: 4.0 ± 1.0 pA, n = 8 cells, N = 2 mice, $P = 0.0002$, two-sided Wilcoxon signed rank test). **e**, Summary of normalized current amplitude (0.61 ± 0.20%, n = 8 cells, N = 2 mice). $^{**}P < 0.005$, $^{***}P < 0.0005$.

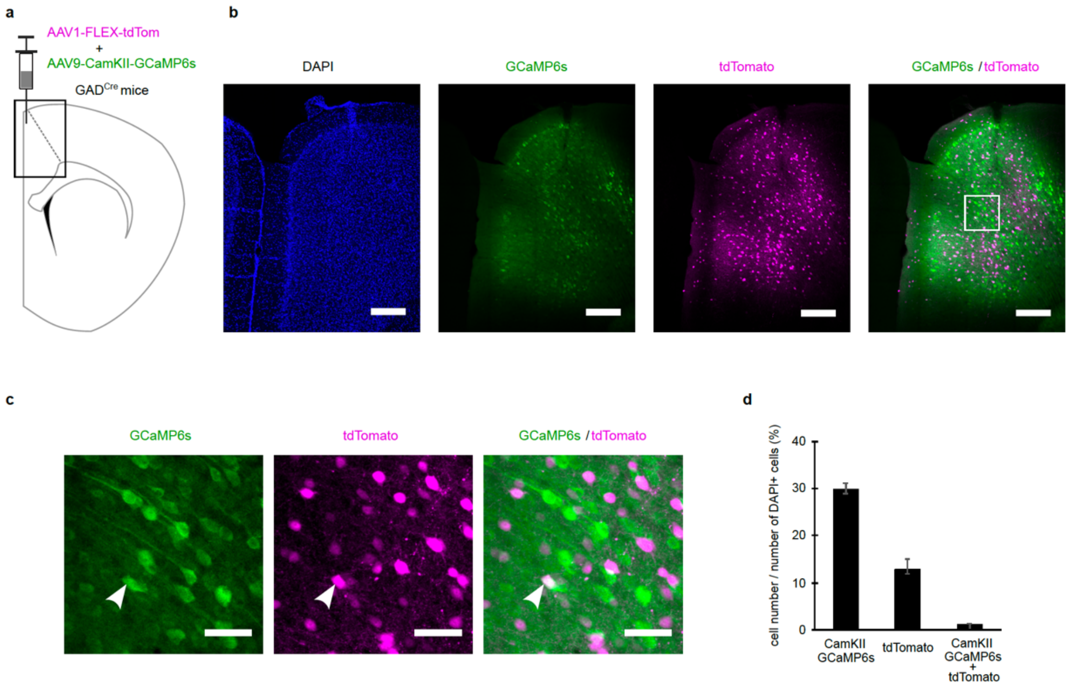

Supplementary Figure 12 | CaMKII-promoter-dependent GCaMP6s expression in the Cg1/2.

a, Schematic evaluating GCaMP6s expression in Cg1/2 neurons in GAD^{Cre} mice. Cre-dependent AAV1-FLEX-tdTomato and non Cre-dependent AAV9-CamKII-GCaMP6s were co-injected into the Cg1/2. **b**, Representative image of CaMKII-promoter-dependent GCaMP6s expression in putative excitatory cells, and Cre-dependent tdTomato expression in Cg1/2 GABAergic cells in a GAD^{Cre} mouse. Scale bar, 200 μm. **c**, Enlarged images of the indicated square area in **b**. Scale bar, 50 μm. **d**, Proportion of GCaMP6s^{+}, tdTomato^{+} and co-labeled GCaMP6^{+}/tdTomato^{+} cells (N = 3 mice, 3 windows (120 μm square)). Data are presented as mean ± S.E.M..

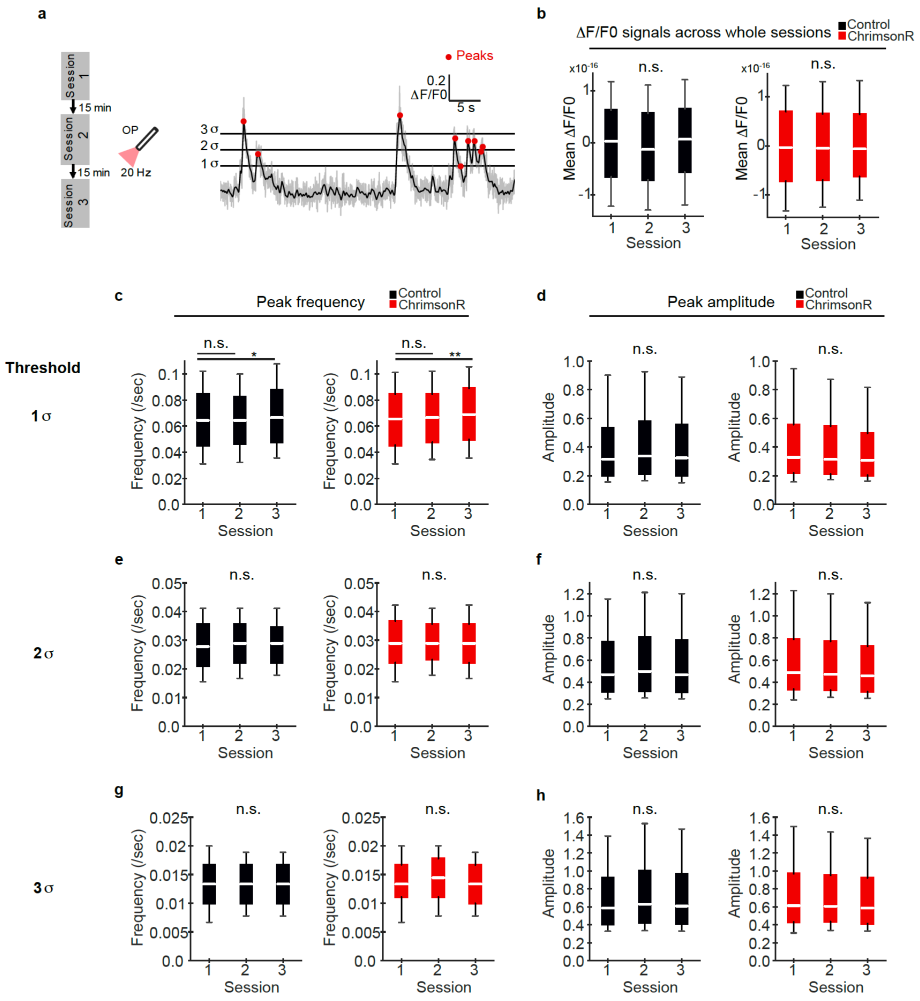

Supplementary Figure 13 | Optogenetic activation of PrL GABAergic projections in the absence of vF stimulation does not change ongoing Ca^{2+} signals in the Cg1/2.

a, Left: Schematic of the procedure used for Ca^{2+} imaging experiments. Right: example of ongoing Ca^{2+} traces and detected peaks from a neuron expressing GCaMP6s in the Cg1/2. Grey line indicates raw ΔF/F0 trace and black line indicates ΔF/F0 trace smoothed with a Gaussian filter (s = 100 ms) for better visualization. Horizontal lines indicate thresholds at one, two and three standard deviation of ΔF/F0. Red circles indicate Ca^{2+} peaks above the indicated standard deviation threshold. **b**, Mean ΔF/F0 in control (left, black) and ChrimsonR (right, red) animals using all acquired frames for session 1, 2 and 3 (each session 15 minutes in duration, control, *P*

= 0.278, ChrimsonR, P = 0.936, Friedman test). **c**, Peak frequencies above the one-, **e**, two- and **g**, three-standard deviation threshold in control (left, black) and ChrimsonR (right, red) mice (1σ, session 1 vs. 2, control, P =1.00, ChrimsonR, P =1.00, session 1 vs. 3, control, P =0.0473, ChrimsonR, P = 0.00873, 2σ, control, P =0.372, ChrimsonR, P =1.00, session 1 vs. 3, control, P =0.333, ChrimsonR, P = 1.00, 3σ, control, P =0.946, ChrimsonR, P =1.00, session 1 vs. 3, control, P =0.916, ChrimsonR, P = 0.0948, $DF_{control}$ = 1253, $DF_{Chrimson}$ = 1423, two-sided Mann-Whitney-U test followed by Bonferroni correction). **d**, Peak amplitudes above the one-, **f**, two- and **h**, three-standard deviation threshold in control (left, black) and ChrimsonR (right, red) mice (1σ, session 1 vs. 2, control, P =0.262, ChrimsonR, P =1.00, session 1 vs. 3, control, P =1.00, ChrimsonR, P = 0.0828, 2σ, control, P =0.400, ChrimsonR, P =1.00, session 1 vs. 3, control, P =1.00, ChrimsonR, P = 0.207, 3σ, control, P =0.482, ChrimsonR, P =1.00, session 1 vs. 3, control, P =1.00, ChrimsonR, P = 0.301, $DF_{control}$ = 1253, $DF_{Chrimson}$ = 1423, two-sided Mann-Whitney-U test followed by Bonferroni correction). Box and whisker plots in **c**-**h** indicate median, interquartile range and 10th to 90th percentiles of the distribution. n.s., not significant, *P < 0.05, **P < 0.01, DF, degree of freedom.

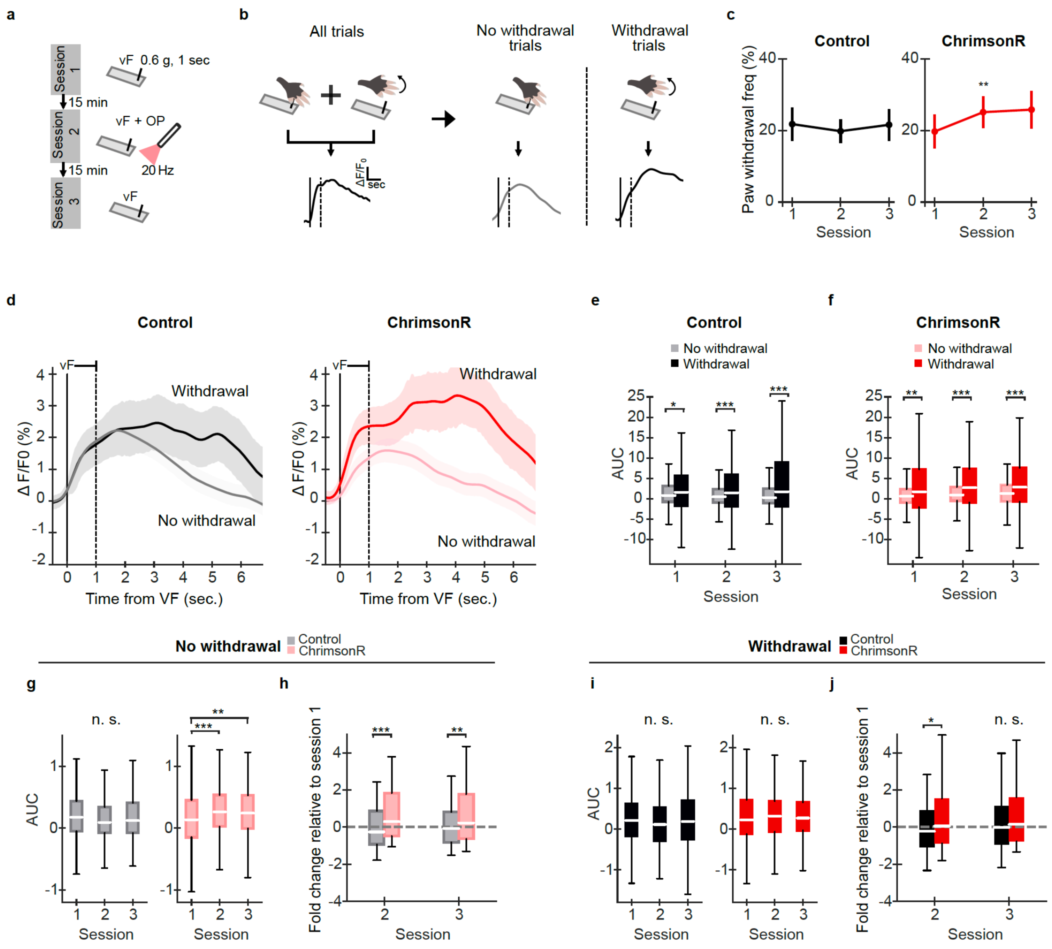

Supplementary Figure 14 | Optogenetic activation of PrL GABAergic projections enhances vF-evoked responses in the Cg1/2 in no-withdrawal trials.

a, Schematic of the procedure used for Ca^{2+} imaging experiments, indicating vF stimulation details and temporal profile of vF and red-light stimulation in the three sessions. **b**, vF-evoked responses in the withdrawal trials and no-withdrawal trials, respectively, are averaged as shown in the schematic. **c**, Paw withdrawal frequency in control (left, N = 13 mice) and in ChrimsonR (right, N = 15 mice) mice (control, $P = 0.943$, ChrimsonR, $P = 0.0437$, Friedman test, session 1 vs. 2, $P = 0.0142$, session 1 vs. 3, $P = 0.339$, two-sided Wilcoxon signed-rank test followed by Bonferroni correction). Data are shown as mean and S.E.M.. **d**, Average neuronal responses to vF stimuli in Cg1/2 neurons during session 1 in withdrawal trials and no-withdrawal trials. Black

vertical line and stippled line indicate onset and end of the vF stimulus, respectively. ΔF/F0 traces were smoothed with a Gaussian filter (s = 250 ms) for visualization purpose. Data are shown as mean and S.E.M.. **e**, Enhanced ΔF/F0 responses in trials with paw withdrawal compared to trials with no paw withdrawal both in control (N = 12 mice, n = 281 neurons, session 1, $P = 0.0338$, session 2, $P = 0.00246$, session 3, $P = 1.63e-05$, DF = 560, two-sided Wilcoxon signed-rank test followed by Bonferroni correction) and **f**, ChrimsonR mice (N = 14 mice, n = 333 neurons, session 1, $P = 0.000958$, session 2, $P = 4.44e-05$, session 3, $P = 1.60e-08$, DF = 664, two-sided Wilcoxon signed-rank test followed by Bonferroni correction). AUC of the vF-evoked response from 0 to 5 second after vF stimulus onset. **g**, Enhanced vF-evoked responses in no-withdrawal trials following optogenetic activation of PrL GABAergic projections. AUC of the vF-evoked response in the Cg1/2 in trials with no paw withdrawal in control (left, grey) and in ChrimsonR (right, pink) mice (control, $P = 0.0819$, ChrimsonR, $P = 0.00797$, Friedman test, session 1 vs. 2, $P = 3.10e-05$, session 1 vs. 3, $P = 0.000940$, DF = 332, two-sided Wilcoxon signed-rank test followed by Bonferroni correction). **h**, Fold change of AUC for session 2 and 3 relative to session 1 in trials with no paw withdrawal (session 2, $P = 1.40e-06$, session 3, $P = 0.00392$, DF = 612, two-sided Mann-Whitney-U test followed by Bonferroni correction). **i**, AUC of the vF-evoked response in the Cg1/2 in trials with paw withdrawal in control (left, black) and in ChrimsonR (right, red) mice (control, $P = 0.0595$, ChrimsonR, $P = 0.357$, Friedman test). **j**, Fold change of AUC for session 2 and 3 relative to session 1 in trials with paw withdrawal (session 2, $P = 0.0171$, session 3, $P = 0.158$, DF = 612, two-sided Mann-Whitney-U test followed by Bonferroni correction). Box and whisker plots in **e-j** indicate median, interquartile range, and 10th to 90th percentiles of the distribution. n.s., not significant, *$P < 0.05$, **$P < 0.01$, ***$P < 0.001$, DF, degree of freedom.

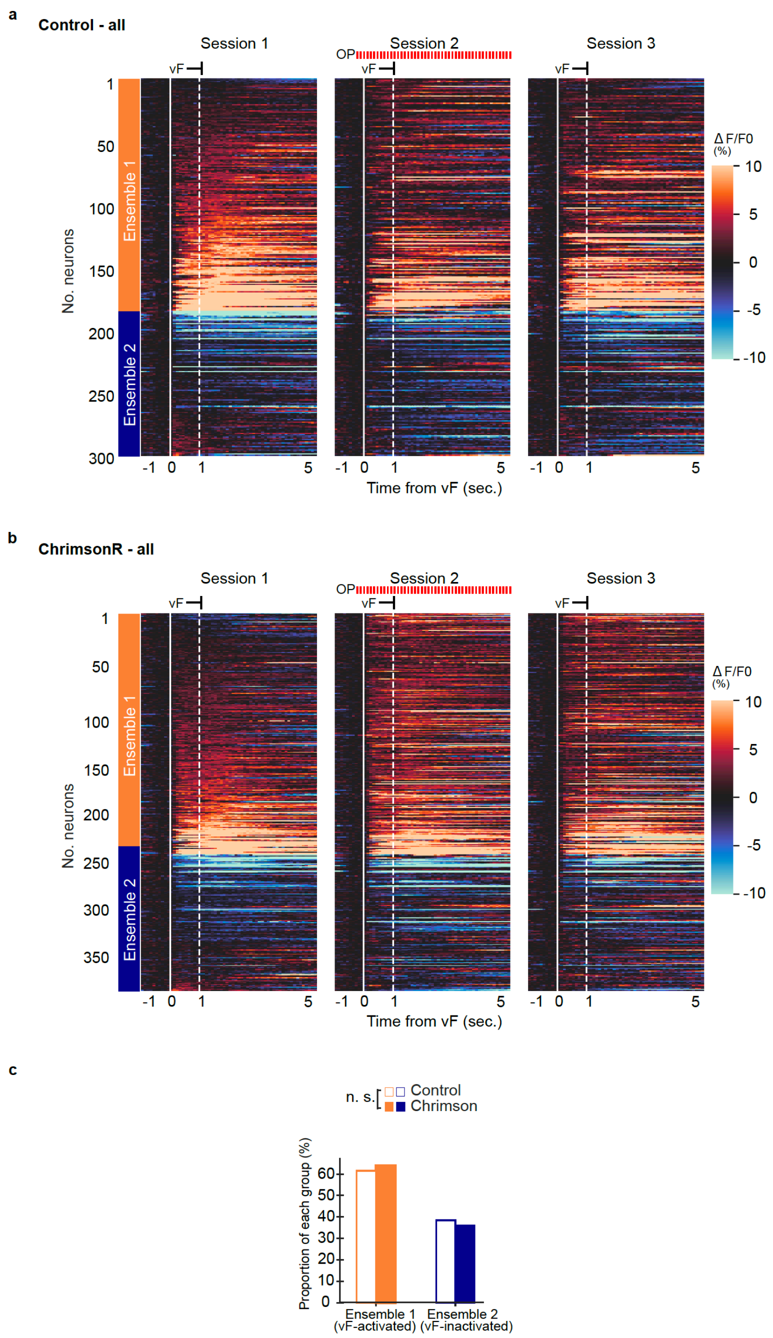

Supplementary Figure 15 | Optogenetic activation of PrL GABAergic projections differentially modulates responses of Cg1/2 neurons.

a, Heatmap indicating mean vF-evoked responses obtained in each session from all neurons of control and **b**, ChrimsonR mice. The responses were sorted based on the maximum value during vF stimulation in session 1. **c**, Overall proportion of vF-activated and -inactivated neurons in

control and ChrimsonR mice ($P = 0.530$, Fisher's exact test). n.s., not significant.

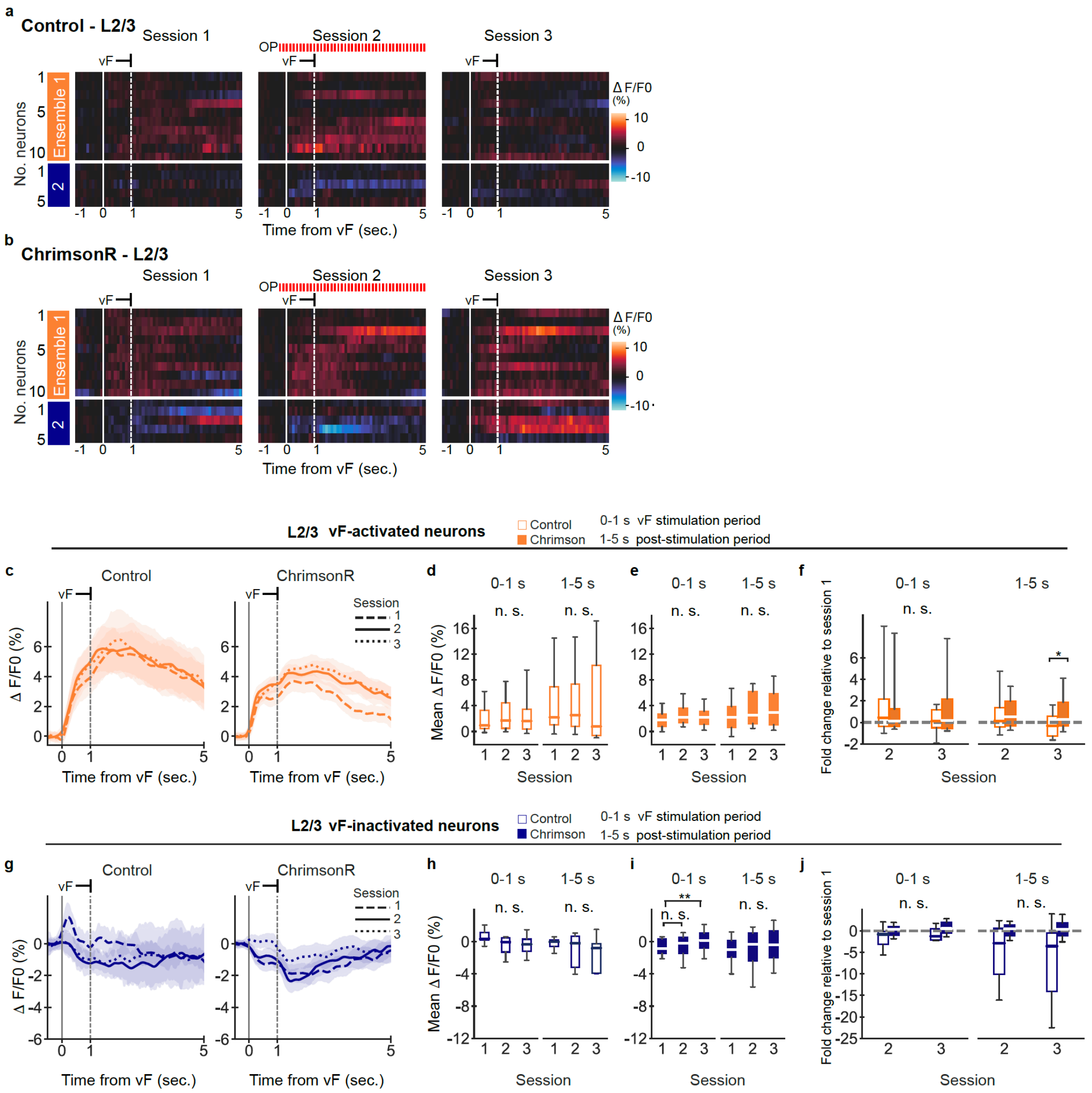

Supplementary Figure 16 | Optogenetic activation of PrL GABAergic projections differentially modulates responses of Cg1/2 L2/3 neurons during and after vF stimulation. **a**, Heatmap indicating mean vF-evoked responses from representative L2/3 neurons of control and **b**, Chrimson mice in each session. Data are assigned to two ensembles based on k-mean clustering. White vertical line and stippled line indicate onset and end of the vF stimulus. The responses were sorted based on the maximum value during vF stimulation in session 1. **c**, Average neuronal responses to vF stimuli of L2/3 vF-activated neurons during session 1 (stippled line), 2

(solid line) and 3 (dotted line) in control (n = 27 neurons) and in ChrimsonR mice (n = 54 neurons). Grey vertical and stippled lines indicate onset and end of vF stimulus, respectively. **d**, Mean ΔF/F0 of L2/3 vF-activated neurons during (0 to 1 sec) and post (1 to 5 sec) vF stimulation in control (open box, during vF stimulation in session 1 vs. 2, $P = 0.654$, session 1 vs. 3, $P = 1.00$, post vF stimulation in session 1 vs. 2, $P = 1.00$, session 1 vs. 3, $P = 1.00$, DF = 26, two-sided Wilcoxon signed-rank test followed by Bonferroni correction) and **e**, in ChrimsonR-expressing mice (filled box, during vF stimulation in session 1 vs. 2, $P = 0.637$, session 1 vs. 3, $P = 1.00$, post vF stimulation in session 1 vs. 2, $P = 0.185$, session 1 vs. 3, $P = 0.0890$, DF = 53, two-sided Wilcoxon signed-rank test followed by Bonferroni correction). **f**, Fold change of ΔF/F0 means for session 2 and 3 relative to session 1 during and post vF stimulation in control (open box) and ChrimsonR mice (filled box, during vF stimulation in session 2, $P = 1.00$, session 3, $P = 1.00$, post vF stimulation in session 2, $P = 1.00$, session 3, $P =0.0450$, DF = 26, two-sided Mann-Whitney-U test followed by Bonferroni correction). **g**, Average neuronal responses to vF stimuli of L2/3 vF-inactivated neurons during session 1 (stippled line), 2 (solid line) and 3 (dotted line) in control (n = 11 neurons) and in ChrimsonR mice (n = 40 neurons). Grey vertical and stippled vertical lines indicate onset and end of vF stimulus, respectively. **h**, Mean ΔF/F0 of L2/3 vF-inactivated neurons during (0 to 1 sec) and post (1 to 5 sec) vF stimulation in control (open box, during vF stimulation in session 1 vs. 2, $P = 0.105$, session 1 vs. 3, $P = 0.853$, post vF stimulation in session 1 vs. 2, $P = 1.00$, session 1 vs. 3, $P = 0.991$, DF = 10, two-sided Wilcoxon signed-rank test followed by Bonferroni correction) and **i**, in ChrimsonR-expressing mice (filled box, during vF stimulation in session 1 vs. 2, $P = 1.00$, session 1 vs. 3, $P = 0.00912$, post vF stimulation in session 1 vs. 2, $P = 1.00$, session 1 vs. 3, $P = 0.788$, DF = 39, two-sided Wilcoxon signed-rank test followed by Bonferroni correction). **j**, Fold change of ΔF/F0 means for session 2 and 3 relative to session 1 during and post vF stimulation in control (open box) and ChrimsonR mice (filled box, during vF stimulation in session 2, $P = 0.416$, session 3, $P = 0.268$, post vF stimulation in session 2, $P = 0.361$, session 3, $P = 0.0733$, DF = 10, two-sided Mann-Whitney-U test followed by Bonferroni

correction). **c**, **g**, ΔF/F0 traces were smoothed with a Gaussian filter (s = 100 ms) for visualization purpose. Data are shown as means ± S.E.M.. **d-f**, **h-j**, box and whisker plots indicate median, interquartile range and 10th to 90th percentiles of the distribution. n.s., not significant, *$P < 0.05$, **$P < 0.01$, ***$P < 0.001$, DF, degree of freedom

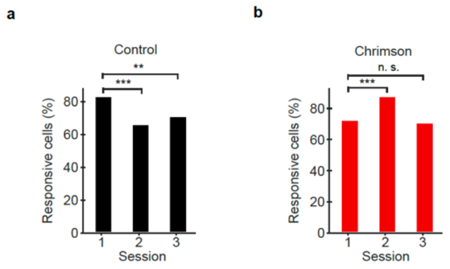

Supplementary Figure 17 | PrL GABAergic projections enhance responsiveness of vF-activated neurons in the Cg1/2.

a, Proportion of vF-activated neurons with significantly altered enhanced Ca^{2+} responses (above 97.5% of shuffled value) to vF stimulation during the three sessions in control mice ($P = 0.000570$, DF = 2, χ^2 test, session 1 vs. 2, $P = 0.000408$, session 1 vs. 3, $P = 0.0130$, Fisher's exact test followed by Bonferroni correction). **b**, Same as in **a**, but for ChrimsonR mice (P = 3.01e-06, DF = 2, χ^2 test, session 1 vs. 2, P = 3.43e-05, session 1 vs. 3, $P = 1.00$, Fisher's exact test followed by Bonferroni correction.). n.s., not significant, *$P < 0.05$, **$P < 0.01$, ***$P < 0.001$, DF, degree of freedom.

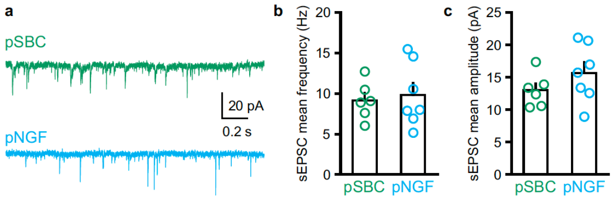

Supplementary Fig. 18 | Mean frequency and mean amplitude of sEPSC in pSBCs and pNGFs are comparable.

a, Representative traces of spontaneous EPSCs (sEPSCs) from a pSBC (top, green) and a pNGF (bottom, blue). **b**, Summary of sEPSC mean frequency (pSBC, n = 6 cells; pNGF, n = 7 cells; N = 3 mice; *P* = 0.945, two-sided Mann-Whitney test). **c**, Summary of sEPSC mean amplitude (pSBC, n = 6 cells; pNGF, n = 7 cells; N = 3 mice; *P* = 0.366, two-sided Mann-Whitney test).

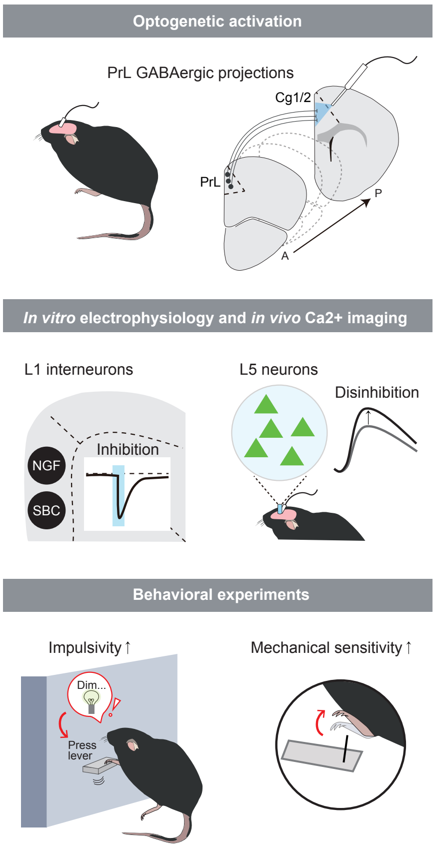

Supplementary Figure 19 | PrL GABAergic projection neurons modulate neuronal activity in the Cg1/2 thereby affecting top-down signaling.

Schematic showing GABAergic projection neurons whose axons are located mainly in layer 1 and that connect the PrL with the Cg1/2. PrL GABAergic projections target L1 INs, namely SBCs and NGFs, and exert a crucial role in regulating the activity in the Cg1/2 by disinhibiting L5 output neurons. The recruitment of these projections increases impulsivity and mechanical sensitivity, behaviors which are known to be modulated by Cg1/2 activity. Abbreviation, PrL, prelimbic cortex, Cg1/2, cingulate area 1 and 2, NGF, neurogliaform cells, SBC, single-bouquet cells, L5, layer 5

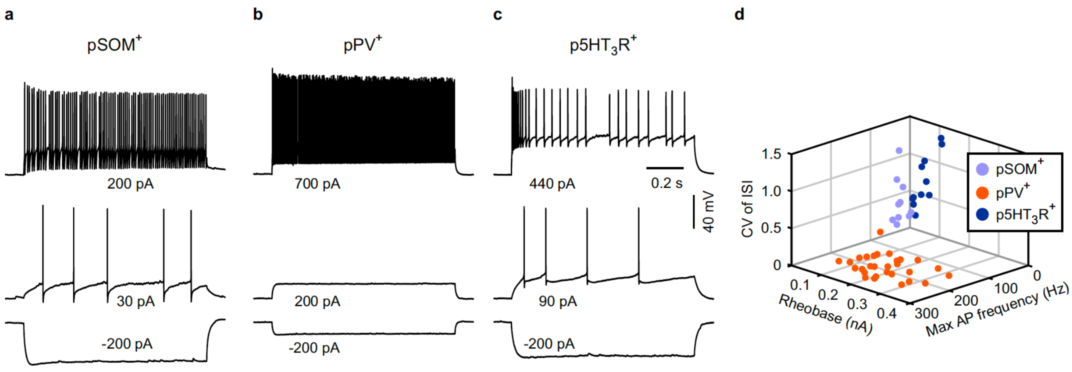

Supplementary Fig. 20 | L 2/3 INs in Cg1/2 exhibit distinct firing patterns.

a, **b**, **c**, Representative voltage responses evoked by the indicated current pulses in a pSOM^{+} cell (**a**), a pPV^{+} cell (**b**) and a p5HT$_3$R^{+} cell (**c**). The pSOM^{+} cell has a low rheobase (-30 pA), the pPV^{+} cell is fast-spiking (211 Hz) and the p5HT$_3$R^{+} cell exhibits an irregular firing pattern (coefficient of variation of interspike interval, CV of ISI: 0.78). **d**, 3D plot of rheobase, max action potential (AP) frequency and CV of ISI for 50 L2/3 INs. Putative SOM^{+} cells (n = 10 cells, N = 6 mice), pPV^{+} cells (n = 29 cells, N = 10 mice) and p5HT$_3$R^{+} cells (n = 11 cells, N = 5 mice) are indicated in pastel blue, navy blue and orange, respectively.

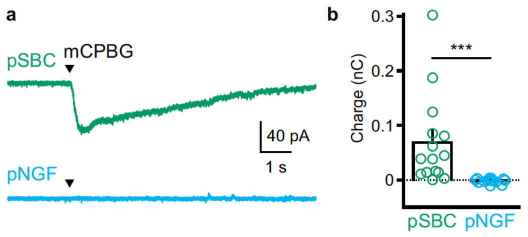

Supplementary Fig. 21 | Putative SBCs, but not pNGF cells, are sensitive to the 5HT3aR agonist.

a, Representative traces of responses evoked by local puffing of 1-(m-chlorophenyl)-biguanide (mCPBG, triangle, 50 µM, 0.1 s) recorded from a pSBC (top, green) and a pNGF (bottom, blue). **b**, Summary of evoked charge (pSBC, n = 15 cells; pNGF, n = 15 cells; N = 3 mice; *P* = 0.000016, ****P* < 0.0005, two-sided Mann-Whitney test). Note that two pSBCs are insensitive to mCPBG.

Supplementary table 1 | Intrinsic electrophysiological properties of L1 INs in Cg1/2

Values are shown as mean ± SEM. Data were analyzed from the dataset of 128 L1 INs (53 pSBCs and 75 pNGFs). For some cells, complete analysis was not available. Therefore, n values here indicate the minimum number of cells analyzed for any given parameter. *P*-values were obtained from two-sided Mann-Whitney test.

Electrophysiological properties	pSBC ($n \geq 48$)	pNGF ($n \geq 49$)	*P* values
Input resistance (MΩ)	403.0 ± 29.1	311.8 ± 12.5	0.041
Membrane time constant (ms)	13.16 ± 0.60	11.99 ± 0.33	0.120
Sag ratio	0.900 ± 0.011	0.937 ± 0.006	0.002
First spike latency (ms)	68.31 ± 4.49	594.6 ± 31.45	7.1×10^{-22}
Depolarizing hump amplitude (mV)	15.16 ± 0.69	1.48 ± 0.34	8.6×10^{-17}
AP amplitude (mV)	74.07 ± 1.22	70.58 ± 1.29	0.025
AP threshold (mV)	-32.87 ± 1.04	-33.74 ± 1.03	0.583
First AHP latency (ms)	4.02 ± 0.72	8.33 ± 0.88	1.3×10^{-6}

Supplementary table 2 | Injection coordinates

Table showing injection coordinates from bregma for the PrL, Cg1/2 and AI.

Injection site	**AP**	**ML**	**DV**
PrL	+2.80 mm	±0.3 mm	-0.9 mm
Cg1/2	+1.20 mm	±0.3 mm	-0.9 mm
AI	+2.20 mm	±2.6 mm	-1.8 mm

Supplementary table 3 | Information of stereotactic injection

Table summarizing information of AAVs, including volume and sites, and transgenic mice used in experiments.

Experiment	Virus	Vendor	Volume (nL)	Mice	Injection site
Anterograde tracing	AAV1-CAG-Flox-eGFP-WPRE-bGH	Penn Vector Core AV-1-ALL854 Addgene #51502	50	GAD^{Cre} or *SOM*^{Cre}	PrL or Cg1/2
Anterograde tracing	AAV1-CaMKIIa-hChR2(h134a)-mCherry	Addgene # 26975	50	GAD^{Cre}	PrL or Cg1/2
Retrograde tracing	Cholera toxin subunit B, Alexa Fluor 555 conjugate	Thermo Fisher Scientific C34776	100	*GAD67*^{EGFP} or *5HT3A*^{EGFP}	PrL or Cg1/2
Retrograde tracing	Retrograde-CAG-AAV-dlox-tdTomato	Zurich Viral Vector Facility v167-retro	100	GAD^{Cre}	AI
Electrophysiological studies	AAV1-EF1a-double floxed-hChR2(H134R)-mCherry	Addgene #20297	100	GAD^{Cre}	PrL
Operant task	AAV1-EF1a-double floxed-hChR2(H134R)-mCherry	Addgene #20297	50	GAD^{Cre}	PrL
Operant task	AAV1-Ef1a-DIO EYFP	Addgene #27056	50	GAD^{Cre}	PrL
Von Frey test	AAV1-EF1a-double floxed-hChR2(H134R)-mCherry	Addgene #20297	100	GAD^{Cre}	PrL
Von Frey test	AAV1-Ef1a-DIO EYFP	Addgene #27056	100	GAD^{Cre}	PrL
In vivo Ca^{2+} imaging	AAV5-Syn-FLEX-rc[ChrimsonR-	Addgene #6723	150	GAD^{Cre}	PrL

	tdTomato]				
In vivo Ca^{2+} imaging	AAV1-FLEX-tdTomato	Addgene #28306	150	GAD^{Cre}	PrL
In vivo Ca^{2+} imaging	AAV9-CamKII.GCaMP6s.WPRE.SV40	Addgene #107790	200	GAD^{Cre}	Cg1/2

Supplementary table 4 | Information of animal numbers used in experiments

Summary of number of mice used in experiments.

Figure	Panel	Number of mice
Fig. 1	m-o	N = 3
Fig. 2	a	N = 3
	b	N = 22
	e	pSBC, N = 4
		pNGF, N = 3
	i, j	N = 4
Fig. 3	c-e	YFP N = 10. ChR2 N = 9
Fig. 4	b - d	YFP N = 9. ChR2 N = 9
Fig. 5	f-k	Control, N = 13
		ChrimsonR, N = 15
Fig. 6	d-k	Control, N = 13
		ChrimsonR, N = 15
Supplementary Fig. 1	g	N = 3
Supplementary Fig. 2	b-d	SOM^{Cre}, N = 3
	f-j	$5HT_{3A}^{EGFP}$, N = 5
Supplementary Fig. 3	m-o	N = 3
Supplementary Fig. 4	g	N = 3
Supplementary Fig. 5	b-d	N = 3
	f-g	N = 3
Supplementary Fig. 6	c	pSBC, N = 16
		pNGF, N = 15
Supplementary Fig. 7	b	Control N = 6. Capsaicin + vF

		N = 6
	d - f	f YFP N = 9 ChR2 N = 9
Supplementary Fig. 8	b<br>c	Control, N = 13<br>ChrimsonR, N = 15
Supplementary Fig. 9	b, d, e	N = 2
Supplementary Fig. 10	d	N = 3
Supplementary Fig. 11	b-h	Control, N = 13<br>ChrimsonR, N = 15
Supplementary Fig. 12	d-j	Control, N = 12<br>ChrimsonR, N = 14
Supplementary Fig. 13	a-c	Control, N = 13<br>ChrimsonR, N = 15
Supplementary Fig. 14	c-j	Control, N = 13<br>ChrimsonR, N = 15
Supplementary Fig. 15		Control, N = 13<br>ChrimsonR, N = 15
Supplementary Fig. 16	b, c	N = 3
Supplementary Fig. 17	d	$pSOM^+$, N = 6<br>pPV^+, N = 10<br>$p5HT_3R^+$, N = 5
Supplementary Fig. 18	b	N = 3
