# Supplementary material for: Long-range inhibition from prelimbic to cingulate areas of the medial prefrontal cortex enhances network activity and response execution
Source: Nat Commun. 2024 Jul 10;15:5772. doi: 10.1038/s41467-024-50055-z (PMC11233578; doi:10.1038/s41467-024-50055-z)
Supplement: Supplementary file 3 — Reporting Summary [file 41467_2024_50055_MOESM3_ESM.pdf]

nature portfolio

Corresponding author(s): Hannah Monyer

Last updated by author(s): May 28, 2024

Reporting Summary

Nature Portfolio wishes to improve the reproducibility of the work that we publish. This form provides structure for consistency and transparency in reporting. For further information on Nature Portfolio policies, see our Editorial Policies and the Editorial Policy Checklist.

Statistics

For all statistical analyses, confirm that the following items are present in the figure legend, table legend, main text, or Methods section.

n/a	Confirmed	
☐	☒	The exact sample size (*n*) for each experimental group/condition, given as a discrete number and unit of measurement
☐	☒	A statement on whether measurements were taken from distinct samples or whether the same sample was measured repeatedly
☐	☒	The statistical test(s) used AND whether they are one- or two-sided<br>*Only common tests should be described solely by name; describe more complex techniques in the Methods section.*
☐	☒	A description of all covariates tested
☐	☒	A description of any assumptions or corrections, such as tests of normality and adjustment for multiple comparisons
☐	☒	A full description of the statistical parameters including central tendency (e.g. means) or other basic estimates (e.g. regression coefficient) AND variation (e.g. standard deviation) or associated estimates of uncertainty (e.g. confidence intervals)
☐	☒	For null hypothesis testing, the test statistic (e.g. *F*, *t*, *r*) with confidence intervals, effect sizes, degrees of freedom and *P* value noted<br>*Give P values as exact values whenever suitable.*
☒	☐	For Bayesian analysis, information on the choice of priors and Markov chain Monte Carlo settings
☒	☐	For hierarchical and complex designs, identification of the appropriate level for tests and full reporting of outcomes
☒	☐	Estimates of effect sizes (e.g. Cohen's *d*, Pearson's *r*), indicating how they were calculated

Our web collection on statistics for biologists contains articles on many of the points above.

Software and code

Policy information about availability of computer code

Data collection	Patch data were collected with PatchMaster version 2x90<br>Operant data were collected using Med PC IV version 4.2<br>Behavioral data during calcium imaging were collected with custom scripts running in Bonsai version 2.4.0
Data analysis	Anatomical data were analyzed with Fiji/ImageJ version 2.0.0.<br>Patch data were analyzed with ClampFit version 10.7 and custom codes running in MATLAB 2020a and Neuromantic version 1.6.3.<br>Patch and behavioral data were analyzed in GraphPad Prism version 5.0 (patch) and 9.0 (behavior).<br>Calcium imaging data were analyzed with CaImAn version 1.8.4, codes in Python 3 (NumPy v1.18.1, SciPy v1.4.1, statsmodels v0.11.1, scikit-learn v0.22.2.post1, pandas v1.0.3, matplotlib v3.2.1, and seaborn v0.10.1.) and Fiji/ImageJ version 2.0.0.

For manuscripts utilizing custom algorithms or software that are central to the research but not yet described in published literature, software must be made available to editors and reviewers. We strongly encourage code deposition in a community repository (e.g. GitHub). See the Nature Portfolio guidelines for submitting code & software for further information.

Data

Policy information about availability of data

All manuscripts must include a data availability statement. This statement should provide the following information, where applicable:

- Accession codes, unique identifiers, or web links for publicly available datasets
- A description of any restrictions on data availability
- For clinical datasets or third party data, please ensure that the statement adheres to our policy

We have provided "Data Availability" statement within Methods section. We have provided all data that are necessary to interpret, verify and extend the research in the article in a Source Data file. In addition, due to the large size of the data (more than ten terabytes), we have indicated that all raw image movies are available on request from the corresponding author.

Research involving human participants, their data, or biological material

Policy information about studies with human participants or human data. See also policy information about sex, gender (identity/presentation), and sexual orientation and race, ethnicity and racism.

Reporting on sex and gender	No human participants or samples were used in this study
Reporting on race, ethnicity, or other socially relevant groupings	No human participants or samples were used in this study
Population characteristics	No human participants or samples were used in this study
Recruitment	No human participants or samples were used in this study
Ethics oversight	No human participants or samples were used in this study

Note that full information on the approval of the study protocol must also be provided in the manuscript.

Field-specific reporting

Please select the one below that is the best fit for your research. If you are not sure, read the appropriate sections before making your selection.

☒ Life sciences ☐ Behavioural & social sciences ☐ Ecological, evolutionary & environmental sciences

For a reference copy of the document with all sections, see nature.com/documents/nr-reporting-summary-flat.pdf

Life sciences study design

All studies must disclose on these points even when the disclosure is negative.

Sample size

No statistical methods were used to predict sample size. The sample sizes reported here were determined on the basis of previous studies.
For anatomical experiments, the sample sizes reported here are comparable to those published in previous studies (Ref 1, 2).
For electrophysiology, the sample sizes reported here are comparable to those published in previous studies (Ref 2, 3, 4).
For behavioral studies, the sample sizes reported here are comparable to those published in previous studies (operant task: Ref 5, 6; vF task and cFos: Ref 7).
For calcium imaging, the sample sizes of neurons obtained in these experiments are comparable to those published in previous studies (Ref 8, 9).

References:

1. Melzer S et al., Cell Reports. 2017
2. Caputi A et al., Neuron. 2022
3. Fuchs EC et al., Neuron. 2016
4. Pouille F, Scanziani M., Nature. 2004
5. Rostron CL et al., BMC Neurosci. 2008
6. van der Veen B et al., Commun Biol. 2021
7. Tan LL et al., Nat Neurosci. 2017

Data exclusions

For all experiments data from mice where either the virus expression, optic fiber placement, or GRIN lens placement were not in the correct region were not included in any analysis. For calcium imaging experiments mismatched ROIs and ROIs that did not correspond to neurons were excluded from the analysis.

Replication

All reported effects were present across many mice and many neurons. Group sizes are shown in the figure legends and results section.

Randomization

Mice were allocated to experimental groups in a random manner.

Blinding	The experimenter was blinded to group identity during data collection.

Reporting for specific materials, systems and methods

We require information from authors about some types of materials, experimental systems and methods used in many studies. Here, indicate whether each material, system or method listed is relevant to your study. If you are not sure if a list item applies to your research, read the appropriate section before selecting a response.

Materials & experimental systems

n/a	Involved in the study
☐	☒ Antibodies
☒	☐ Eukaryotic cell lines
☒	☐ Palaeontology and archaeology
☐	☒ Animals and other organisms
☒	☐ Clinical data
☒	☐ Dual use research of concern
☒	☐ Plants

Methods

n/a	Involved in the study
☒	☐ ChIP-seq
☒	☐ Flow cytometry
☒	☐ MRI-based neuroimaging

Antibodies

Antibodies used	Chicken polyclonal anti-EGFP Thermo Fisher Scientific CAT# A10262; RRID: AB_2534023<br>Alexa 488 conjugated donkey anti-chicken Jackson ImmunoResearch Laboratories CAT# 703-545-155; RRID: AB_2340375<br>Rabbit polyclonal anti-DsRed Clontec Living Colors CAT# 632496; RRID: AB_10013483<br>Cy3 conjugated donkey anti-rabbit Jackson ImmunoResearch Laboratories CAT# 711-165-152; RRID: AB_2307443<br>Alexa 647-conjugated anti-rabbit IgG Invitrogen CAT# A-31573; RRID: AB_2536183<br>rabbit anti-c-Fos abcam CAT# ab190289<br>4,6-diamidino-2-phenylindole (DAPI) Invitrogen, D1306
Validation	All antibodies used in this study were commercially available and the validation information, including relevant references, can be found at the supplier's website.<br>Chicken polyclonal anti-EGFP [https://www.thermofisher.com/antibody/product/GFP-Antibody-Polyclonal/A10262]<br>Alexa 488 conjugated donkey anti-chicken [https://www.jacksonimmuno.com/catalog/products/703-545-155]<br>Rabbit polyclonal anti-DsRed Clontec Living Colors [https://www.takarabio.com/products/antibodies-and-elisa/fluorescent-protein-antibodies/red-fluorescent-protein-antibodies]<br>Cy3 conjugated donkey anti-rabbit [https://www.jacksonimmuno.com/catalog/products/711-165-152]<br>Alexa 647-conjugated anti-rabbit IgG [https://www.thermofisher.com/antibody/product/Donkey-anti-Rabbit-IgG-H-L-Highly-Cross-Adsorbed-Secondary-Antibody-Polyclonal/A-31573]<br>rabbit anti-c-Fos [https://www.abcam.com/en-de/products/primary-antibodies/c-fos-antibody-bsa-free-ab190289]<br>4,6-diamidino-2-phenylindole (DAPI) [https://www.thermofisher.com/order/catalog/product/de/de/D1306]

Animals and other research organisms

Policy information about studies involving animals; ARRIVE guidelines recommended for reporting animal research, and Sex and Gender in Research

Laboratory animals	8-14 week old male GADcre (Fuchs et al., 2001)), GAD67EGFP (Tamamaki et al., 2003), SOMCre (Melzer et al., 2012), PVCre (Hippenmeyer et al., 2005), VIPCre (Taniguchi et al., 2011), 5HT-EGFP (Inta et al., 2008) mice maintained on a C57/BL6N background.
Wild animals	This study did not involve wild animals.
Reporting on sex	All experiments were conducted on male mice.
Field-collected samples	This study did not involve field-collected samples.
Ethics oversight	All experiments were approved by the Regierungspraesidium Karlsruhe, Germany (AZ 35-9185.81/G-119/14, 35-9185.81/G-157/16, 35-9185.81/G-61/18, 35-9185.81/G-131/19) in compliance with the European guidelines for the care and use of laboratory animals.

Note that full information on the approval of the study protocol must also be provided in the manuscript.

Plants

Seed stocks	*Report on the source of all seed stocks or other plant material used. If applicable, state the seed stock centre and catalogue number. If plant specimens were collected from the field, describe the collection location, date and sampling procedures.*
Novel plant genotypes	*Describe the methods by which all novel plant genotypes were produced. This includes those generated by transgenic approaches, gene editing, chemical/radiation-based mutagenesis and hybridization. For transgenic lines, describe the transformation method, the number of independent lines analyzed and the generation upon which experiments were performed. For gene-edited lines, describe the editor used, the endogenous sequence targeted for editing, the targeting guide RNA sequence (if applicable) and how the editor was applied.*
Authentication	*Describe any authentication procedures for each seed stock used or novel genotype generated. Describe any experiments used to assess the effect of a mutation and, where applicable, how potential secondary effects (e.g. second site T-DNA insertions, mosiacism, off-target gene editing) were examined.*
